# Lethality caused by ADP-glucose accumulation is suppressed by salt-induced carbon flux redirection in cyanobacteria

**DOI:** 10.1093/jxb/erz559

**Published:** 2019-12-20

**Authors:** Sandra Díaz-Troya, Miguel Roldán, Manuel J Mallén-Ponce, Pablo Ortega-Martínez, Francisco J Florencio

**Affiliations:** 1 Instituto de Bioquímica Vegetal y Fotosíntesis, Universidad de Sevilla-CSIC, Sevilla, Spain; 2 University of Cambridge, UK

**Keywords:** ADP-glucose, cyanobacteria, energy charge, glucosylglycerol, nitrogen metabolism, photosynthesis, *Synechocystis*

## Abstract

Cyanobacteria are widely distributed photosynthetic organisms. During the day they store carbon, mainly as glycogen, to provide the energy and carbon source they require for maintenance during the night. Here, we generate a mutant strain of the freshwater cyanobacterium *Synechocystis* sp. PCC 6803 lacking both glycogen synthases. This mutant has a lethal phenotype due to massive accumulation of ADP-glucose, the substrate of glycogen synthases. This accumulation leads to alterations in its photosynthetic capacity and a dramatic decrease in the adenylate energy charge of the cell to values as low as 0.1. Lack of ADP-glucose pyrophosphorylase, the enzyme responsible for ADP-glucose synthesis, or reintroduction of any of the glycogen synthases abolishes the lethal phenotype. Viability of the glycogen synthase mutant is also fully recovered in NaCl-supplemented medium, which redirects the surplus of ADP-glucose to synthesize the osmolite glucosylglycerol. This alternative metabolic sink also suppresses phenotypes associated with the defective response to nitrogen deprivation characteristic of glycogen-less mutants, restoring the capacity to degrade phycobiliproteins. Thus, our system is an excellent example of how inadequate management of the adenine nucleotide pools results in a lethal phenotype, and the influence of metabolic carbon flux in cell viability and fitness.

## Teaser

Lack of glycogen synthases results in the lethal accumulation of their substrate, ADP-glucose, but providing an alternative metabolic sink restores cell viability and the nutrient deprivation response.

## Introduction

Cyanobacteria are prokaryotic organisms that are widely distributed among almost every habitat on Earth. They are especially important in aquatic ecosystems as primary producers and N_2_ fixers, introducing carbon and nitrogen into the trophic chains. These organisms also had a crucial role in the history of the planet: oxygenic photosynthesis, which releases O_2_ as a by-product, evolved in ancient cyanobacteria and played a key role in the transition from the primitive anaerobic and reducing atmosphere to the aerobic and oxidizing environment that exists today (Lyons *et al.*, 2014; Soo *et al.*, 2017). A cyanobacterial ancestor also gave rise to the plastids in modern photosynthetic eukaryotes by an endosymbiotic process (Ponce-Toledo *et al.*, 2017). Cyanobacteria also have promising biotechnological potential in sustainable bioproduction, in which solar-driven atmospheric CO_2_ removal by photosynthetic fixation can be coupled to the synthesis of commodities and chemicals of interest (Woo, 2017).

**Fig. 1.**
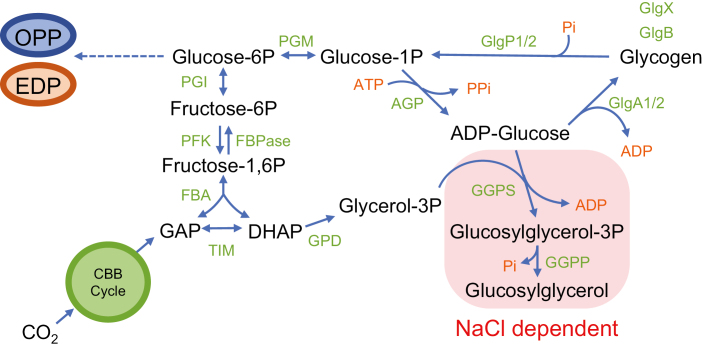
Scheme of metabolic pathways for the synthesis of glycogen and glucosylglycerol in *Synechocystis*. AGP, ADP-glucose pyrophosphorylase; EDP, Entner–Doudoroff pathway; FBA, fructose bisphosphate aldolase; FBPase, fructose bisphosphatase; GGPP, glucosylglycerolphosphate phosphatase; GGPS, glucosylglycerolphosphate synthase; GlgA1/2, glycogen synthases; GlgB, glycogen branching enzyme; GlgP1/2, glycogen phosphorylases; GlgX, glycogen debranching enzyme; GPD, glycerol-3-phosphate dehydrogenase; OPP, oxidative pentose phosphate pathway; P, phosphate; PFK, phosphofructokinase; PGI, Glucose-6-phosphate isomerase; PGM, phosphoglucomutase; TIM, triosephosphate isomerase. (This figure is available in colour at *JXB* online.)

In cyanobacteria, photosynthetically fixed CO_2_ can be incorporated into amino acids, for protein synthesis and growth, and into a wide range of other organic compounds, including storage polysaccharides, that serve as a source of carbon and energy when required, mainly in light/dark regimes. Most cyanobacteria accumulate glycogen as a storage polysaccharide, although starch or starch-like polysaccharides have also been found in a few cyanobacteria (Nakamura *et al.*, 2005; Suzuki *et al.*, 2013). Glycogen synthesis requires the action of several enzymes (Fig. 1), including ADP-glucose pyrophosphorylase (AGP), which generates ADP-glucose (ADP-Glc) from glucose-1-phosphate and ATP, and glycogen synthases (GlgA), which add ADP-Glc to the non-reducing end of the growing glycogen molecule. In the model freshwater cyanobacterium *Synechocystis* sp. PCC 6803 (hereafter *Synechocystis*) there is one gene, *glgC*, that codes for AGP, and two genes, *glgA1* and *glgA2*, coding for GlgA1 and GlgA2, respectively. Glycogen accumulates during the day and is consumed overnight. Glycogen accumulation also increases when cells enter the stationary phase and, mainly, as part of the adaptation to limitation of nutrients such as nitrogen (Schwarz and Forchhammer, 2005). The response to nitrogen deprivation also includes other metabolic and physiological changes in the cells to prepare them for these unfavorable conditions, such as modifications of the photosynthetic machinery in a process known as bleaching or chlorosis (Forchhammer and Schwarz, 2019). During bleaching, the pigmented phycobiliproteins that form the phycobilisome—the main light-harvesting antenna for photosynthesis in cyanobacteria—are degraded, and the color of the cell changes from blue-green to yellowish. This process allows the nitrogen present in these proteins to be recycled as a supply to adapt to the environmental nitrogen limitation. Prolonged nitrogen deficiency results in the cells entering a dormant-like state in which they can survive for extended periods of time (Forchhammer and Schwarz, 2019).

Mutants impaired in glycogen accumulation have a pleiotropic phenotype. In addition to reduced viability in periods of darkness (Gründel *et al.*, 2012), they are unable to develop an adequate response to nitrogen deprivation. *glgC* mutant cells do not show phycobiliprotein degradation (and so maintain their blue-green color) and stop dividing immediately after nitrogen removal, in contrast to wild-type cells, which divide once. These mutants also have a metabolite overflow phenotype, excreting organic acids, mainly pyruvate and 2-oxoglutarate, in a process that has been suggested to act as a compensation for the lack of the carbon buffer function of glycogen (Carrieri *et al.*, 2012; Gründel *et al.*, 2012; Cano *et al.*, 2018).

In addition to the accumulation of sugars as storage polysaccharides, cyanobacteria also accumulate sugars with an osmoprotective function. Sucrose, trehalose, glucosylglycerol (GG), or glycine betaine can be found as osmoprotectants in cyanobacteria in habitats with various saline concentrations (Klähn and Hagemann, 2011). In response to salt stress, *Synechocystis* synthetizes both sucrose and GG. In the synthesis of GG (Fig.1), glucosylglycerol-phosphate synthase catalyzes the formation of the intermediate glucosylglycerol-phosphate from ADP-Glc and glycerol-3-phosphate, and glucosylglycerol-phosphate phosphatase dephosphorylates this intermediate to produce GG (Klähn and Hagemann, 2011). Thus, the synthesis of both glycogen and GG requires ADP-Glc as a precursor metabolite. *Synechocystis* mutants defective in the synthesis of GG show a phenotype of salt sensitivity (Marin *et al.*, 1998).

Here, we report that the lack of both GlgA1 and GlgA2 is lethal for *Synechocystis* because it results in a massive accumulation of ADP-Glc, which, in turn, causes photosynthetic defects and a strong decline in the cell energy charge. Cultivation of these cells in salt-supplemented medium provides an alternative metabolic destination for ADP-Glc, redirecting it to the synthesis of GG, which allows the cells to remain viable. Thus, lack of GlgA activity converts *Synechocystis* into a halodependent cyanobacterium. In addition, redirection of the metabolic carbon flux to this alternative sink allows recovery of the bleaching process and mitigation of the metabolite overflow in a mutant lacking glycogen. This demonstrates that carbon flux, and not glycogen accumulation itself, is required for progression of the bleaching process in response to nitrogen deprivation.

## Materials and methods

### Culture conditions

*Synechocystis* cells were cultivated in BG11C [BG11 (Rippka *et al.*, 1979) supplemented with 1 g l^−1^ NaHCO_3_] in conical flasks bubbled with a stream of 1% (v/v) CO_2_ in air, under continuous illumination (4000 K LED lights, 75–85 µmol m^−2^ s^−1^) at 30 °C. Trace amounts of copper in BG11C-Cu were chelated with bathocuproinedisulfonic acid at a final concentration of 1 µM. When required, BG11C was supplemented with 0.5 M NaCl. For plate cultures, the media were supplemented with 1% (w/v) Bacto agar (Difco) and antibiotics were added when required (50 µg ml^−1^ kanamycin, 20 µg ml^−1^ chloramphenicol, 2.5 µg ml^−1^ spectinomycin, 2.5 µg ml^−1^ streptomycin, and 50 µg ml^−1^ nourseothricin). Cell growth was monitored spectrophotometrically, following the absorbance of the cultures at 750 nm (OD_750_). For NaCl-removal experiments, exponentially growing cells (typically at OD_750_≈0.7–1.5) in NaCl-supplemented BG11C were harvested by centrifugation (4,300 *g* for 5 min), washed once in the corresponding medium, and resuspended in NaCl-supplemented or standard BG11C to OD_750_=0.5. In nitrogen-deprivation experiments, harvested cells were washed twice in nitrogen-free medium (BG11_0_C) with or without 0.5 M NaCl supplementation to ensure complete removal of nitrogen before resuspending the cells in NaCl-supplemented or standard BG11_0_C (OD_750_=0.5).

### Generation of mutant strains

The sequences of the genes studied were obtained from CyanoBase (http://genome.microbedb.jp/cyanobase/). These genes are *glgC* (gene ID slr1176), *glgA1* (gene ID *sll0945*), *glgA2* (gene ID *sll1393*), *glnN* (gene ID *slr0288*), *petJ* (gene ID *sll1796*), *adk* (gene ID *sll1815*), *purA* (gene ID *sll1823*), and *ggpS* (gene ID *sll1566*).

Strains used in this work are described in Table 1, and primers are listed in Supplementary Table S1 at *JXB* online. To generate the triple mutant *glgC*, *glgA1*, *glgA2* (SGLG06 strain), ΔAGP was sequentially transformed with the plasmid pΔglgA1-Km (Díaz-Troya *et al.*, 2014) to delete *glgA1* (rendering SGLG04) and then with pΔglgA2-Sp (Díaz-Troya *et al.*, 2014) to delete *glgA2* (SGLG06).

**Table 1.** Strains used in this work

Name	Parental strain	Genotype	Reference
**WT**		Wild-type *Synechocystis* sp. PCC 6803	
**ΔAGP**	WT	Δ*glgC::*C.C1	(Díaz-Troya *et al.*, 2014)
**ΔglgA1**	WT	Δ*glgA1::*C.K1	(Díaz-Troya *et al.*, 2014)
**ΔglgA2**	WT	Δ*glgA2::*SpΩ	(Díaz-Troya *et al.*, 2014)
**SGLG04**	ΔAGP	Δ*glgC::*C.C1, Δ*glgA1::*C.K1	This work
**SGLG06**	SGLG04	Δ*glgC::*C.C1, Δ*glgA1::*C.K1, Δ*glgA2::*SpΩ	This work
**SGLG14**	ΔAGP	Δ*glgC::*C.C1, *glnN::PpetJ:glgC:*Nat^R^	This work
**SGLG15**	SGLG06	Δ*glgC::*C.C1, Δ*glgA1::*C.K1, Δ*glgA2::*SpΩ, *glnN::PpetJ:glgC:*Nat^R^	This work
**SGLG36**	ΔglgA2	Δ*glgA1::*C.K1, Δ*glgA2::*SpΩ	This work
**SGLG37**	SGLG36	Δ*glgA1::*C.K1, Δ*glgA2::*SpΩ, Δ*glgC::*C.C1	This work
**SGLG49**	SGLG36	*glgA1:*C.C1, Δ*glgA2::*SpΩ	This work
**SGLG50**	SGLG36	Δ*glgA1::*C.K1, *glgA2:*C.C1	This work
**SGLG51**	SGLG49	*glgA1:*C.C1, *glgA2:*Nat^R^	This work

ΔAGP and SGLG06 were used as parental strains to achieve the Cu-regulated expression of *glgC* (SGLG14 and SGLG15 strains) by transforming them with the pGLG51 plasmid. To construct this plasmid, the coding sequence of *glgC* was amplified from *Synechocystis* genomic DNA with the primers glgC_NdeI_5′ and glgC_NotI_3′, and inserted in the *Nde*I/*Not*I site in the p*glnN*_P*petJ*_NatT plasmid. This latter plasmid contains a synthetic polylinker that allows cloning and expression of genes under the regulation of the *petJ* gene promoter, and includes the sequence for homologous recombination in the *glnN* locus and a Nat resistance cassette conferring nourseothricin resistance (Giner-Lamia *et al.*, 2015).

The SGLG36 strain was generated by deleting the *glgA1* gene with the pΔglgA1-Km plasmid in the ΔglgA2 parental strain. To generate the SGLG37 strain, SGLG36 was used as the background and the entire *glgC* open reading frame (ORF) was deleted with the pΔglgC-Cm(+) plasmid (Díaz-Troya *et al.*, 2014).

Glycogen synthases were restored in SGLG36 by reintroducing *glgA1* (SGLG49), *glgA2* (SGLG50), or both (SGLG51). The pGLG87 plasmid was used to obtain the SGLG49 strain. Primers glgA1_UP_5′/glgA1_BamHI_UP2_3′ and glgA1_BamHI_DO2_5′/glgA1_DO_3′were used in a two-step PCR to amplify the complete *glgA1* ORF plus 500 nucleotides upstream and downstream and to introduce a *Bam*HI site after the ORF. The PCR product was cloned into the pGEM-T plasmid and a chloramphenicol resistance cassette was introduced in the *Bam*HI site. The SGLG50 strain was generated with the pGLG89 plasmid. This plasmid was constructed following a similar strategy, with a two-step PCR using primers glgA2_UP_5′/glgA2_BamHI_UP2_3′ and glgA2_BamHI_DO2_5′/glgA2_DO_3′ to amplify the *glgA2* ORF plus 500 nucleotides upstream and downstream. A *Bam*HI site was also generated next to the stop codon. After cloning this PCR product into pGEM-T, a chloramphenicol resistance cassette was introduced in the *Bam*HI site. To generate the SGLG51 strain, SGLG49 was used as the background and *glgA2* was reintroduced using the plasmid pGLG85. This plasmid is similar to pGLG89 but contains a nourseothricin resistance cassette in the *Bam*HI site instead of a chloramphenicol resistance cassette.

All the constructions were designed for single integration by double recombination. Strains were segregated in the presence of the appropriate antibiotics in BG11C plates, except for the SGLGL36 strain, which also required the addition of 250mM NaCl.

### Cell extract preparation and western blotting

Cells grown in liquid culture were collected by centrifugation (4300 *g* for 10 min), resuspended in 50 mM Tris–HCl (pH 8), 25 mM NaCl, and 1 mM phenylmethylsulphonyl fluoride, and lysed using glass beads in a Mini-Beadbeater (BioSpec Products) by two cycles of vortexing for 1 min followed by 5 min on ice. After centrifugation (15 000 *g* for 30 min at 4 °C), the soluble fraction was recovered and proteins were quantified with the Bradford assay (Bio-Rad). For western blotting, proteins were resolved by SDS-PAGE and transferred to a nitrocellulose membrane (BioRad). Blots were blocked in blocking solution [5% non-fat dry milk (AppliChem PanReac) in PBS–Tween 20] and incubated with polyclonal antibodies to *Synechocystis* GlgC (1:20 000 dilution), GlgA1 (1:5000 dilution), GlgA2 (1:5000 dilution) (all described in Díaz-Troya *et al.*, 2014), and TrxA (1:5000 dilution) (Navarro *et al.*, 2000) in blocking solution. Horseradish peroxidase-conjugated secondary antibodies (Sigma, 1:25 000 dilution in blocking solution) and ECL Prime Western Blotting Detection Reagent (GE Healthcare) were used to visualize the immunoblots.

### Glucosylglycerol and nucleotides determination

GG was determined according to Schoor *et al.* (1995) with modifications. In brief, 4 ml aliquots of cell cultures were centrifuged (15 000 *g* for 2 min) and GG was extracted from the cell pellets by incubation at 65 °C for 4 h in 1 ml 80% ethanol. Extracts were centrifuged (15 000 *g* for 5 min) and supernatants were collected and dried in a vacuum centrifuge. Pellets were dissolved in 100 µl ultrapure water and filtered (0.20 µm, Millex-GN Nylon, Millipore). Samples (10 µl) were analyzed by HPLC in a Waters LC Module I Plus system equipped with a Waters 410 Differential Refractometer detector using an Aminex HPX87H Column (BioRad), operated in isocratic mode (5 mM H_2_SO_4_, 0.6 ml min^−1^).

To determine adenine nucleotides and the nucleotide-sugar ADP-Glc, 25 OD_750_ equivalent cells were collected, immediately resuspended to a final volume of 225 µl in 0.33 M perchloric acid, and left on ice for 10 min. After centrifugation for 5 min at 15 000 *g* and 4 °C, 150 µl of the supernatant was transferred to a new tube, neutralized with 25 µl 2.5 M KOH and 0.5 M MES, and centrifuged as above. Neutralized supernatants were filtered (10K Amicon® Ultra 0.5 ml Centrifugal Filters, Millipore) and 10 µl aliquots of supernatant were injected in a SUPELCOSIL™ LC-18-T HPLC Column (Supelco) fitted to a Hitachi ELITE LaChrom System. Analyzed metabolites were detected at 254 nm (Deuterium lamp, Hitachi) with a Diode Array Detector L-2455.

### Extracellular pyruvate and 2-oxoglutarate determination

For determination of extracellular metabolites, 10 ml culture samples were centrifuged (4000 *g*, 10 min, 4 °C), and supernatants were filtered (Mixed cellulose ester membrane, 0.45 µm, HAWP, Millipore) to remove any remaining cellular material, lyophilized (VirTis BenchTop Pro Freeze dryer, SP Scientific), and stored at –80 °C until analyzed. Samples were resuspended in 1 ml H_2_O and 50–200 µl aliquots were analyzed by enzymatic coupled assays. For determination of pyruvate, samples were assayed in a reaction mixture containing 375 mM Tris–HCl (pH 7.5) and 0.11 mM NADH, and the reaction was initiated by the addition of 5 mU lactate dehydrogenase (Sigma). For determination of 2-oxoglutarate, the reaction mixture contained 325 mM Tris–HCl (pH 7.5), 375 mM NH_4_Cl, and 0.11 mM NADH, and the reaction was initiated with 12 mU glutamate dehydrogenase (Sigma). In both cases, NADH oxidation was followed spectrophotometrically at 340 nm, and the metabolite concentration was calculated by comparison with standard curves.

### Oxygen evolution

Oxygen evolution was measured in a Clark-type O_2_ electrode (Hansatech) at 30 °C. Cells were harvested at different times and adjusted to OD_750_=0.5 with BG11C. Just before the measurements were made, 10 mM NaHCO_3_ was added. For O_2_ evolution at growth light intensity and light saturation curves, light was provided by an LED light source (LED1, Hansatech) and light intensity was controlled by an Oxylab+ control unit (Hansatech).

### Pulse-amplitude-modulation fluorometry

Photosynthetic parameters were determined by pulse-amplitude-modulation fluorometry with a DUAL-PAM-100 (Walz). Photosystem II (PSII) operating efficiency [Y(II)] and PSII maximum efficiency (*F*_v_′/*F*_m_′) were calculated as (*F*_m_′–*F*_s_)/*F*_m_′ and (*F*_m_′–*F*_0_′)/*F*_m_′ _,_ respectively (Stirbet *et al.*, 2019). Cells were adapted to dark for 10 min before fluorescence measurements were made. Values for maximal fluorescence (*F*_m_′) were determined after 5 min of repeated saturation pulses in the presence of actinic light. *F*_s_ was measured during exposure to actinic light. Minimum fluorescence (*F*_0_′) was obtained after switching off the actinic light.

### RNA isolation and northern blot analysis

Total RNA isolation was performed by vortexing cell pellets from 40 ml cultures in the presence of acid-washed baked glass beads (0.25–0.3 mm diameter) and phenol–chloroform. Isolated RNA was quantified with a NanoDrop 1000 (Thermo Scientific), separated by electrophoresis on 1.2% agarose denaturing formaldehyde gels, and blotted on to nylon membranes (Immobilon-NY+, Millipore) (Sambrook and Russell, 2001). Prehybridization, hybridization, and washing steps were performed following the manufacturer’s recommendations. Probes for northern blot hybridization were obtained by PCR with oligonucleotide pairs sll1815_1F/sll1815_1R for *adk*, sll1823_1F/sll1823_1R for *purA*, and sll1566_1F/sll1566_1R for *ggpS* (sequences listed in Supplementary Table S1) and random-prime labeled (Rediprime II, GE Healthcare) with α-[^32^P] dCTP. Hybridization signals were detected and analyzed with a Cyclone Plus Storage Phosphor system (Perkin-Elmer). The signal for *rnpB* was used for normalization.

## Results

### A mutant lacking both glycogen synthases is not viable under standard growth conditions

**Fig. 2.**
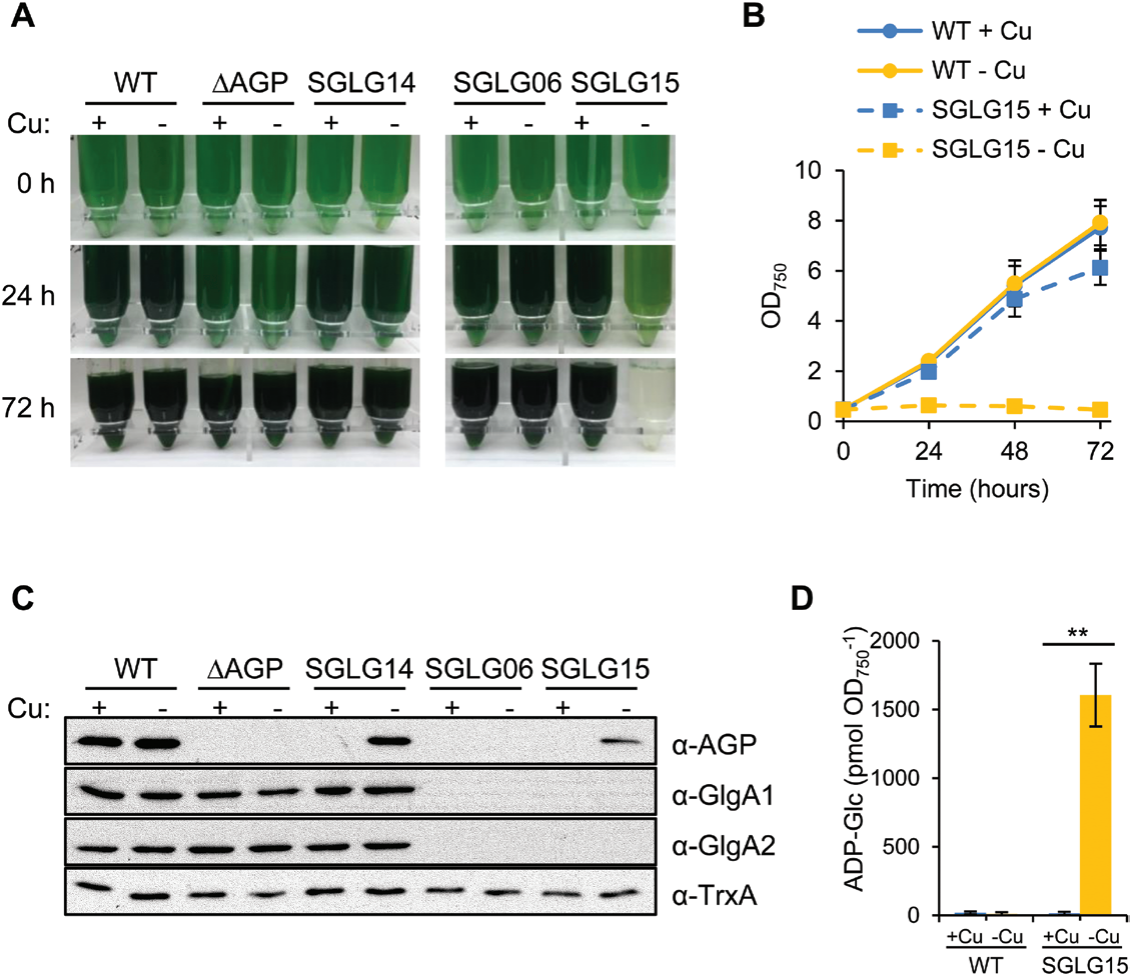
Regulated expression of GlgC in a strain lacking both glycogen synthases (SGLG15) induces growth arrest. WT, ∆AGP, SGLG14, SGLG06, and SGLG15 cells were grown in BG11C with or without copper to repress (+Cu) or activate (-Cu) the P*petJ*-driven expression of *glgC* in SGLG14 and SGLG15. (A) Photographs of the strains. (B) Growth curves. For clarity, only WT and SGLG15 are displayed. (C) Western blot analysis of total soluble extracts with the indicated antibodies. TrxA was used as loading control. (D) Measurement of intracellular ADP-Glc content 24 h after the initiation of the experiment. **P<0.01 (unpaired two-tailed Student’s *t* test). Data in (B) and (D) are means ±SEM from three biological replicates. (This figure is available in colour at *JXB* online.)

The viability of glycogen-deficient mutant cyanobacteria is well documented in the literature. In fact, AGP mutant strains have been generated independently by several laboratories (Miao *et al.*, 2003; Suzuki *et al.*, 2010; Carrieri *et al.*, 2012; Gründel *et al.*, 2012; Guerra *et al.*, 2013; Hickman *et al.*, 2013; Díaz-Troya *et al.*, 2014; Li *et al.*, 2014; van der Woude *et al.*, 2014; Benson *et al.*, 2016; Namakoshi *et al.*, 2016; Kodama *et al.*, 2018). However, we and others were unable to obtain fully segregated glycogen-deficient *Synechocystis* strains lacking both glycogen synthases in the presence of *glgC* (Yoo *et al*., 2014, 2015). This was not the case when a *glgC* mutant was used as the parental strain. In this genetic background, the *glgA1* and *glgA2* genes were deleted and the resulting *glgC*, *glgA1*, *glgA2* triple mutant [SGLG06 strain (Δ*glgC::*C.C1, Δ*glgA1::*C.K1, Δ*glgA2::*SpΩ); Table 1, Supplementary Fig. S1) was viable (Fig. 2A). This finding suggests that the lack of both GlgA1 and GlgA2 in combination with the presence of AGP generates alterations additional to those caused by the lack of glycogen polymer. To confirm this, we generated a functional *glgA1*, *glgA2* double mutant by introducing, in the SGLG06 background, the *glgC* gene under the control of the *petJ* promoter [SGLG15 strain (Δ*glgC::*C.C1, Δ*glgA1::*C.K1, Δ*glgA2::*SpΩ, *glnN::PpetJ:glgC:*Nat^R^); Table 1, Supplementary Fig. S1]. In this strain, *glgC* expression was regulated by the presence of copper in the medium, being repressed in the presence of copper and active in its absence. When we grew SGLG15 in copper-replete medium, it showed a wild-type (WT) growth phenotype and no AGP protein was detected (Fig. 2A–C). The removal of copper from the medium induced the expression of *glgC*, and AGP accumulated in the absence of GlgA1 and GlgA2 (Fig. 2C), generating a functional *glgA1*, *glgA2* double mutant. This caused a dramatic phenotype: cell growth ceased immediately and cells died in 48–72 h (Fig. 2A, B). This phenotype was not attributable to a toxic effect of copper deficiency, as the changes in the content of copper in the culture medium did not affect the growth of WT or SGLG06 strains (Fig. 2A, B). In addition, regulated expression of *glgC* did not cause any effect in a *glgC* mutant background if the *glgA1* and *glgA2* genes were present [SGLG14 (Δ*glgC::*C.C1, *glnN::PpetJ:glgC:*Nat^R^); Table 1, Supplementary Fig. S1, Fig. 2A, C]. In these growth conditions, the presence of AGP in a mutant lacking glycogen synthases was lethal for the cells.

**Fig. 3.**
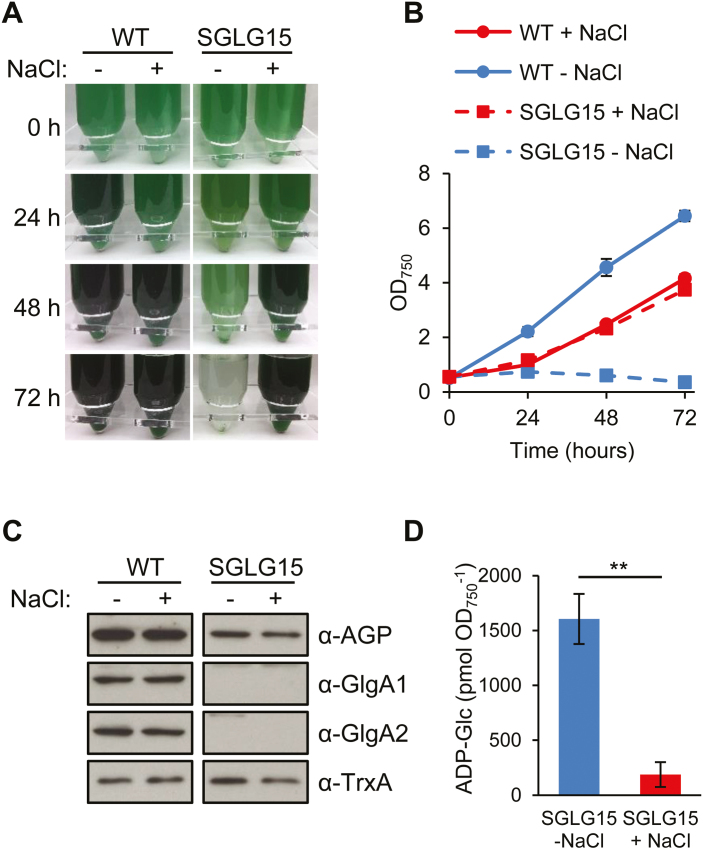
NaCl rescues the growth phenotype of the SGLG15 strain expressing GlgC. WT and SGLG15 cells were grown in BG11C without copper to allow P*petJ*-driven expression of *glgC* in SGLG15. (A) Photographs and (B) growth curves of WT and SGLG15 in the presence or absence of 0.5 M NaCl. (C) Western blot analysis with the indicated antibodies of total soluble extracts from WT and SGLG15 cells cultivated in the presence or absence of 0.5 M NaCl. TrxA was used as loading control. (D) Measurement of intracellular ADP-Glc content in SGLG15 24 h after transfer to BG11C without copper in the presence or absence of 0.5 M NaCl. **P<0.01 (unpaired two-tailed Student’s *t* test). Data are means ±SEM from four (B) or three (D) biological replicates. (This figure is available in colour at *JXB* online.)

As glycogen synthesis by GlgA1 and GlgA2 is the main destination of the ADP-Glc synthesized by AGP, we next hypothesized that the expression of AGP in SGLG15 leads to the accumulation of ADP-Glc, and that this accumulation is toxic for the cells. To test this hypothesis, we first measured the levels of ADP-Glc in WT and SGLG15 in the presence and absence of copper in the medium. High ADP-Glc levels were detected in SGLG15 only in the absence of copper, that is, when AGP expression was induced in a background lacking *glgA1* and *glgA2* (Fig. 2D). In order to test the second part of the hypothesis, we designed a strategy to decrease the level of ADP-Glc. ADP-Glc is also the substrate of glucosylglycerol-phosphate synthase, the key enzyme involved in the synthesis of osmoprotectant GG in response to salt stress (Hagemann and Erdmann, 1994) (Fig. 1). Thus, salt stress would induce the synthesis of GG and reduce the accumulation of ADP-Glc, reducing its toxic effect. The use of medium supplemented with 0.5 M NaCl for the culture of the AGP-expressing SGLG15 strain was associated with a 16-fold decrease in the amount of ADP-Glc and rescued the lethal phenotype of SGLG15 in the absence of copper (Fig. 3). We can conclude that the accumulation of ADP-Glc was responsible for the lethal phenotype of the SGLG15 strain when *glgC* is expressed.

### Generation of a glycogen synthase double mutant in NaCl-supplemented medium

The results described above showed that a functional GlgA double mutant was viable in NaCl-supplemented medium. Hence, we used this growth condition in the selection procedure and obtained completely segregated colonies of the GlgA double mutant lacking *glgA1* and *glgA2* [SGLG36 strain (Δ*glgA1::*C.K1, Δ*glgA2::*SpΩ); Table 1, Supplementary Fig. S1, Supplementary Fig. S2A, B]. As the triple mutant lacking GlgA1, GlgA2, and AGP (SGLG06) was perfectly viable (Fig. 2A), we analyzed the levels of these proteins by western blot to rule out the possibility that colonies with lower or no expression of AGP were selected during the segregation procedure. None of the glycogen synthases were detected in SGLG36, but, in contrast to SGLG06, AGP levels were similar to those found in the WT strain (Supplementary Fig. S2C). In addition, maintenance of this mutant strain on plates was strictly dependent on the presence of NaCl in the medium (Supplementary Fig. S2D). Thus, lack of GlgA1 and GlgA2 in the presence of AGP transforms the freshwater cyanobacterium *Synechocystis* into a strictly salt-dependent organism.

### Accumulation of ADP-Glc leads to cell growth arrest

**Fig. 4.**
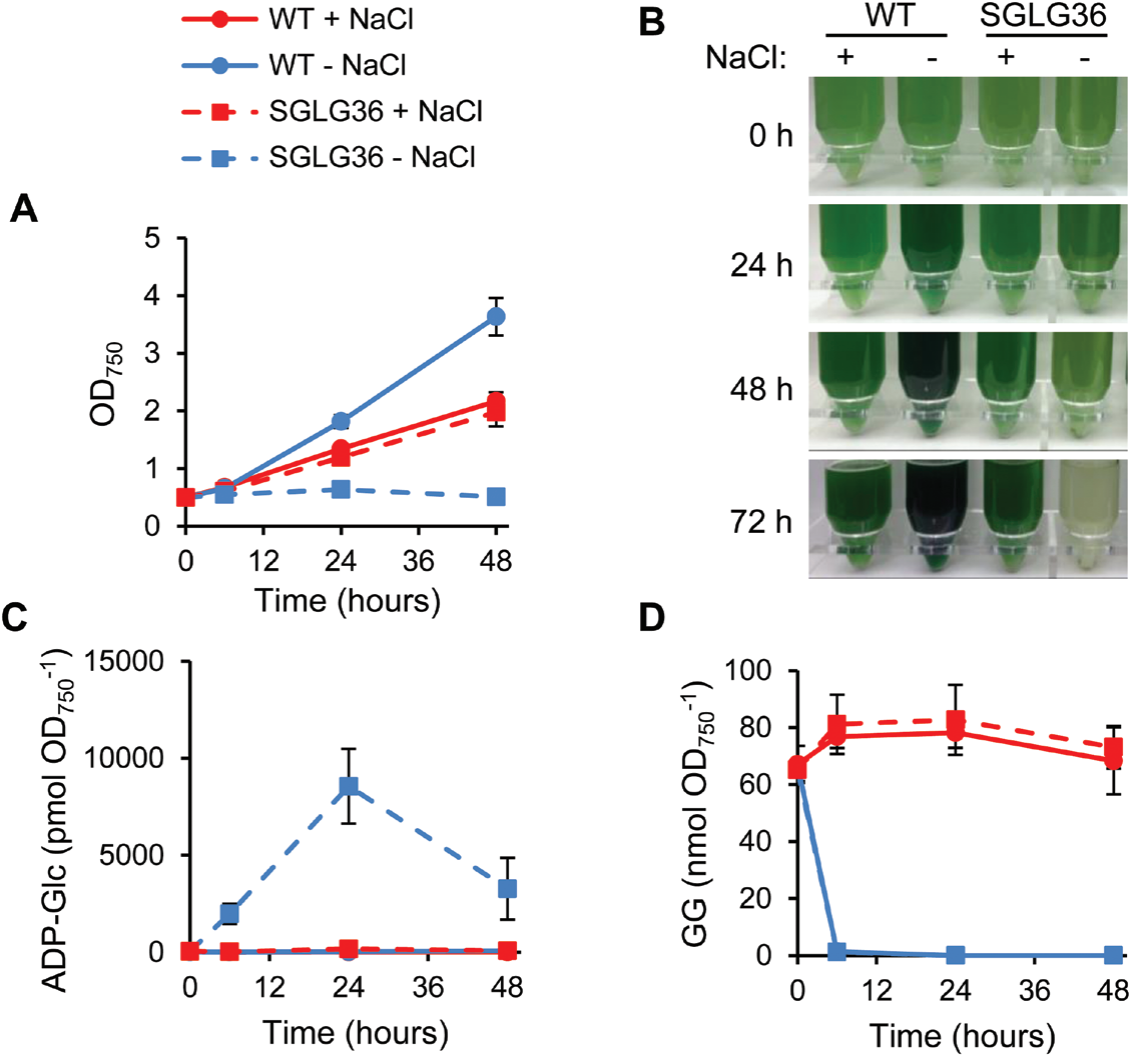
The SGLG36 mutant can be rescued by redirecting ADP-Glc to GG synthesis. WT and SGLG36 cells were grown in BG11C containing NaCl and transferred to medium with or without 0.5 M NaCl. (A) Growth curves and (B) photographs of WT and SGLG36 strains. (C) Intracellular ADP-Glc content. (D) Glucosylglycerol content. Data are means ±SEM from four (A, C) or three (D) biological replicates. (This figure is available in colour at *JXB* online.)

To further understand the salt-dependent phenotype of SGLG36, we analyzed the growth of cells cultivated in medium containing 0.5 M NaCl and then transferred to medium with or without 0.5 M NaCl by centrifugation and resuspension in the appropriate medium. WT and SGLG36 cultures in NaCl-supplemented medium showed similar growth (Fig. 4A, B). However, when transferred to standard NaCl-free medium, SGLG36 cells, but not WT cells, displayed complete growth arrest, and the cultures became white within 2–3 days after the shift (Fig. 4A, B). The effect of NaCl on the growth of the SGLG36 strain was dose dependent: 50 mM NaCl was enough to prevent cells from dying, and increasing concentrations of NaCl up to 0.5 M improved cell growth (Supplementary Fig. S3). Measurement of ADP-Glc indicated that the transfer of SGLG36 to standard NaCl-free medium produced a rapid accumulation of ADP-Glc up to 8 nmol ADP-Glc OD_750_^−1^ (Fig. 4C); by contrast, ADP-Glc was almost undetectable in the WT growing in either NaCl-supplemented or standard medium. As expected, both WT and SGLG36 cells grown in NaCl-supplemented medium synthesized GG, reaching values of 77.9±4.1 and 84.0±12.0 nmol GG OD_750_^−1^ for WT and SGLG36, respectively. Furthermore, after removal of NaCl, the amount of GG rapidly decreased to non-detectable levels in both strains (Fig. 4D). These results clearly indicate that the principal metabolic destination for ADP-Glc is glycogen synthesis by glycogen synthases, and consequently lack of both *glgA1* and *glgA2* resulted in high accumulation of ADP-Glc, provoking a severely impaired growth phenotype. ADP-Glc accumulation can be alleviated by redirecting the pool of ADP-Glc towards GG synthesis in NaCl-containing medium, which restores normal cell growth.

**Fig. 5.**
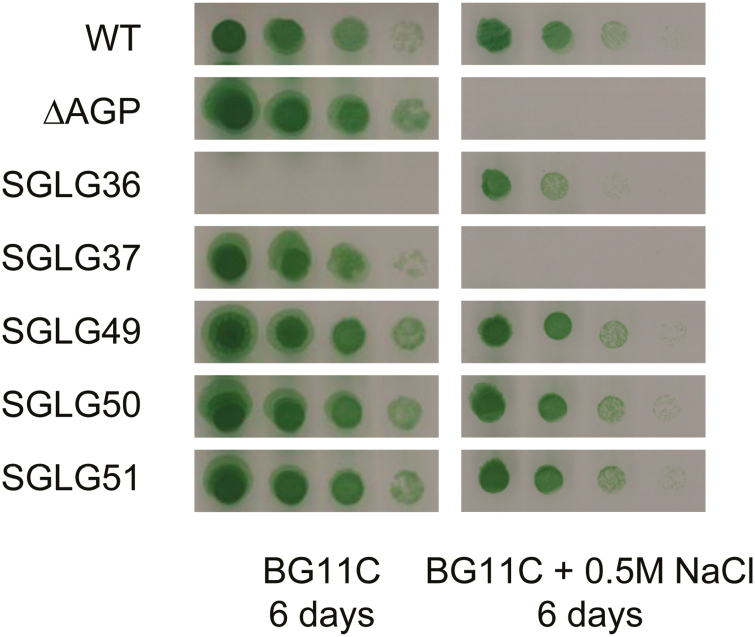
Prevention of ADP-Glc accumulation by restoring glycogen synthesis or by the deletion of *glgC* suppresses the salt dependence of SGLG36. Cells were grown to mid-exponential phase in BG11C (except SGLG36 cells, which were grown in BG11C+0.5 M NaCl) and adjusted to OD_750_=0.5. Serial culture dilutions were spotted on to BG11C plates with or without 0.5 M NaCl and incubated under continuous light for 6 days. (This figure is available in colour at *JXB* online.)

Genetic modifications in the SGLG36 background to abolish or minimize the accumulation of ADP-Glc suppressed the salt dependence of this strain. Thus, elimination of the capacity for ADP-Glc synthesis by the deletion of *glgC* [SGLG37 strain (Δ*glgA1::*C.K1, Δ*glgA2::*SpΩ, Δ*glgC::*C.C1); Table 1, Supplementary Fig. S1], or restoration of the capacity for glycogen synthesis by the reintroduction of *glgA1* [SGLG49 strain (*glgA1:*C.C1, Δ*glgA2::*SpΩ)], *glgA2* [SGLG50 strain (Δ*glgA1::*C.K1, *glgA2:*C.C1)], or both genes [SGLG51 strain (*glgA1:*C.C1, *glgA2:*Nat^R^); Table 1, Supplementary Fig. S1] rescued the growth phenotype in standard medium (Fig. 5). As expected, the lack of ADP-Glc in the ∆AGP and SGLG37 mutants impaired GG synthesis and consequently prevented their growth in NaCl-supplemented medium (Fig. 5).

### Accumulation of ADP-Glc negatively affects photosynthesis

**Fig. 6.**
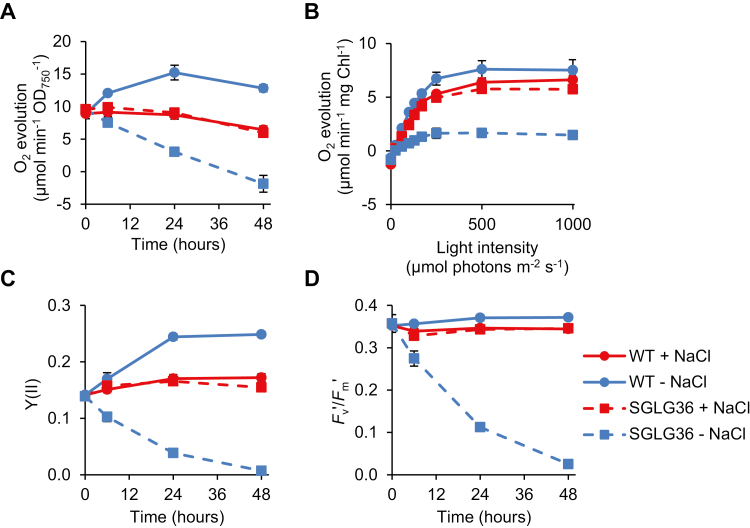
Photosynthetic parameters are altered in the mutant strain SGLG36. WT and SGLG36 cells were grown in BG11C containing NaCl and transferred to medium with or without 0.5 M NaCl. (A) Photosynthetic O_2_ evolution rates under growth light intensity (100 µmol photons m^−2^ s^−1^) measured on a Clark-type O_2_ electrode normalized to cell density. (B) Light saturation curves normalized to chlorophyll content measured 24 h after the start of the experiment. (C) PSII operating efficiency [Y(II)]. (D) PSII maximum efficiency (*F*_v_′/*F*_m_′). Data are means ±SEM from three biological replicates. (This figure is available in colour at *JXB* online.)

To gain insight into the cause of the lethal phenotype of the SGLG36 strain in the absence of salt, we evaluated its photosynthetic activity, as the rate of O_2_ evolution, under growth light intensity (100 µmol photons m^−2^ s^−1^) at different times after NaCl removal. When cultivated in NaCl-supplemented medium, the net O_2_ evolution normalized to cell density was similar in WT and SGLG36 (Fig. 6A). However, removal of NaCl decreased the photosynthetic activity of SGLG36 as soon as 6 h after exchange of the medium, with a minimum activity at 48 h when no net O_2_ evolution was detected (Fig. 6A). By contrast, in the WT cell cultures removal of salt promoted an increase in O_2_ evolution (Fig. 6A).

In addition, a thorough characterization of the photosynthetic capacity of these strains was carried out by generating light saturation curves. In NaCl-supplemented medium, the saturation curves were similar in WT and SGLG36, reaching maximum O_2_ evolution rates at 500 µmol photons m^−2^ s^−1^ (Fig. 6B). However, 24 h after removal of NaCl, the light saturation curve of SGLG36 saturated at a lower light intensity than WT (~250 µmol photons m^−2^ s^−1^ in SGLG36 versus 500 µmol photons m^−2^ s^−1^ in WT) and reached a 3-fold lower maximum O_2_ evolution rate than WT (Fig. 6B). In accordance with the O_2_ evolution data, measurements of chlorophyll fluorescence by pulse-amplitude-modulation fluorometry showed that PSII operating efficiency [Y(II)] and PSII maximum efficiency (*F*_v_′/*F*_m_′) strongly decreased in SGLG36 within 24 h after salt removal (Fig. 6C, D). These results indicate that in the absence of an alternative sink for ADP-Glc, effective photosynthetic activity and maximum photosynthetic capacity are impaired in SGLG36.

### Accumulation of ADP-Glc alters the adenylate energy charge

**Fig. 7.**
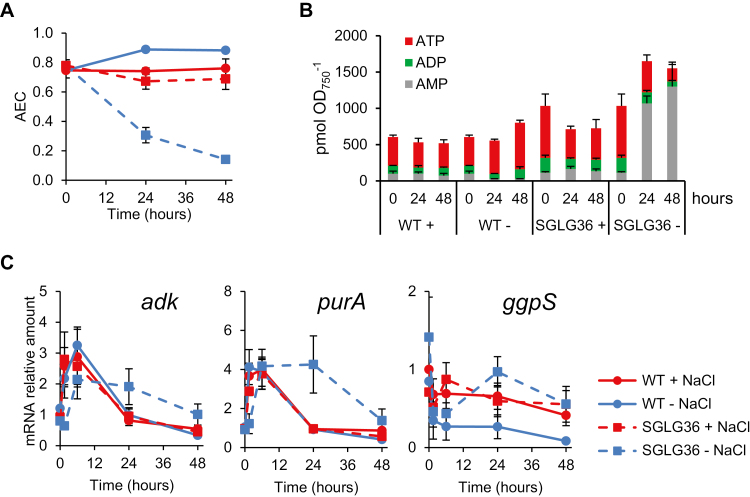
Energy status and transcriptional response to NaCl removal are altered in the SGLG36 mutant. WT and SGLG36 cells were grown in BG11C containing NaCl and transferred to medium with or without NaCl. (A) Adenylate energy charge (AEC). (B) Levels of adenine nucleotides. (C) Quantification of the relative mRNA levels of *adk*, *purA*, and *ggpS*. Radioactive signals were quantified and normalized to the *rnpB* signal. Data are means ±SEM from three (A, B) or four (C) biological replicates.

Adenylate energy charge (AEC) is representative of the energy state of the cell and is a major factor in the regulation of the metabolism (Atkinson, 1968). As defined by Atkinson (1968), AEC is calculated as ([ATP]+0.5×[ADP])/([ATP]+[ADP]+[AMP]). In metabolically active cells this value is kept between 0.70 and 0.95 (De La Fuente *et al.*, 2014). To determine whether the massive sequestration of ADP as ADP-Glc was leading to an imbalance in the AEC of the cell, we quantified the amounts of adenine nucleotides in WT and SGLG36 cells cultivated in NaCl-supplemented or standard NaCl-free medium. The AEC was similar in WT and SGLG36 cells grown in NaCl-supplemented medium, with a value close to 0.8 (Fig. 7A). While the AEC in WT cells increased slightly after the removal of NaCl, a significant decrease was observed in the AEC of SGLG36 cells 24 h after salt removal, reaching values as low as 0.1 after 48 h (Fig. 7A). The imbalance in AEC was due not only to a decrease of the ATP pool but especially to a strong increase of the AMP pool, which reached levels in standard medium 10 times as high as in NaCl-supplemented medium. This made the total adenylate pool (ATP+ADP+AMP) higher in SGLG36 after salt removal than in any other strain and condition (Fig. 7B). Thus, these data suggest that the dramatic phenotype of SGLG36 in standard culture medium is mostly due to an extremely low energy charge, incompatible with the maintenance of normal cell metabolism.

### Transcriptional response to the imbalance in cellular energy charge

Maintenance of an adequate adenine nucleotide equilibrium is essential for metabolic processes and growth, and adenylate kinase (encoded by *adk*) plays an important role in this equilibrium (Igamberdiev and Kleczkowski, 2015). This enzyme catalyzes the formation of ADP at the expense of AMP and ATP, and its activity could compensate for a shortage of ADP sequestered as ADP-Glc in SGLG36 cells under standard conditions. Furthermore, *de novo* synthesis of AMP also requires the enzyme adenylosuccinate synthetase, encoded by *purA* (Stayton *et al.*, 1983). To assess whether there was a transcriptional response in SGLG36 to the imbalance in AEC, we analyzed the expression of *adk* and *purA*. After the transfer of WT cells to fresh medium with or without NaCl, the expression of *adk* and *purA* was transiently induced before returning to basal levels (Fig. 7C). A similar expression pattern was observed in SGLG36 cells transferred to NaCl-supplemented medium. However, the expression of *adk* and *purA* in the SGLG36 strain remained high for up to 24 h after salt removal (Fig. 7C). These results agree with the high levels of AMP found in SGLG36 and suggest that there is a transcriptional response to compensate for the sequestration of ADP as ADP-Glc.

Changes in salt conditions—and hence GG synthesis—are also accompanied by changes in the expression of *ggpS*, coding for glucosylglycerol-phosphate synthase, the main enzyme in GG synthesis (Marin *et al.*, 2002). In WT cells, removal of NaCl switched off the expression of *ggpS* (Fig. 7C). Surprisingly, the expression of *ggpS* did not show the expected pattern in SGLG36 cells after transfer to standard medium. In the absence of NaCl, the expression of *ggpS* decreased transiently but was induced again after 6 h, peaking at 24 h , even when no GG could be detected in those cells (Fig. 4D, Fig. 7C). These results indicate that although the SGLG36 cell responds transcriptionally to the accumulation of ADP-Glc, this is not enough to avoid its lethal effect.

### Suppression of the characteristic phenotype in response to nitrogen deprivation in a glycogen-deficient mutant

**Fig. 8.**
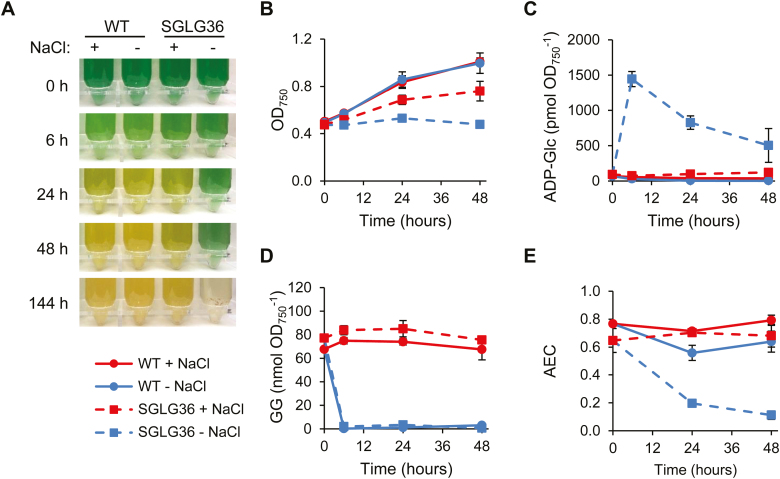
The SGLG36 mutant bleaches in response to nitrogen deprivation when grown in medium containing NaCl. WT and SGLG36 cells were grown in BG11C (17.6 mM NaNO_3_) containing 0.5 M NaCl and then transferred to BG11_0_C (nitrogen-free) medium with or without 0.5 M NaCl. (A) Photographs and (B) growth curves of WT and SGLG36 strains. (C) Intracellular ADP-Glc and (D) glucosylglycerol contents. (E) Adenylate energy charge (AEC). Data are means ±SEM from four (A), three (C, E), or two (D) biological replicates. (This figure is available in colour at *JXB* online.)

Glycogen-deficient mutants have been generated in *Synechocystis* and other cyanobacteria by the inactivation of *glgC* or *glgA*. These mutants display a characteristic phenotype during nitrogen deprivation: they immediately stop dividing, do not bleach, and excrete organic acids (Carrieri *et al.*, 2012; Gründel *et al.*, 2012). The ultimate cause of this phenotype is not well understood, but it implies the role of glycogen as the main energy and carbon buffer. The SGLG36 mutant represents a very interesting tool to address this question, since it provides the opportunity to test whether glycogen synthesis itself is strictly necessary to respond to nitrogen deprivation or whether the presence of an alternative carbon sink, such as GG synthesis, could alleviate the phenotype described for these mutants. Thus, we investigated WT and SGLG36 strains subjected to nitrogen deprivation. The strains were grown in NaCl-supplemented BG11C and nitrogen was removed while maintaining or removing the NaCl. As indicated by the increase in OD_750_, WT cells were able to divide once in the absence of nitrogen, as previously described (Li and Sherman, 2002), independently of the presence of NaCl in the medium. As expected, there was no increase in the OD_750_ of the SGLG36 culture when NaCl and nitrogen were removed from the medium at the same time (Fig. 8A, B). These cells accumulated ADP-Glc rapidly and their AEC decreased, reaching values close to 0.1 after 48 h (Fig. 8C, E). This was mainly due to an increase in the level of AMP (Supplementary Fig. S4). However, this non-growth phenotype in nitrogen-depleted medium was partially suppressed by the presence of 0.5 M NaCl (Fig. 8A, B), which efficiently redirected ADP-Glc to the synthesis of GG (Fig. 8C, D).

The second main characteristic of the phenotype of glycogen-defective mutants in nitrogen-deprivation conditions is their inability to degrade phycobiliproteins. To evaluate the degradation of the phycobilisomes, we performed whole-cell absorbance spectra. As shown in Supplementary Fig. S5, the peak at 620–625 nm, corresponding to phycobilin absorption, decreased in nitrogen-starved WT cultures independently of the presence of NaCl in the medium, rendering the cultures a yellowish color (Fig. 8A). A similar bleaching phenotype was observed in nitrogen-starved SGLG36 cultures in the presence of NaCl (Fig. 8A, Supplementary Fig. S5). In the absence of NaCl, SGLG36 cells retained a green color, similar to what has been described for AGP mutant cells (Gründel *et al.*, 2012), until they finally died (Fig. 8A).

**Fig. 9.**
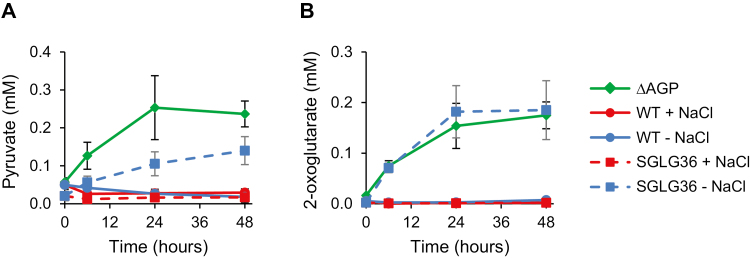
The SGLG36 mutant does not excrete organic acids in response to nitrogen deprivation when grown in a medium containing NaCl. WT and SGLG36 cells were grown in BG11C (17.6 mM NaNO_3_) containing NaCl and transferred to BG11_0_C (nitrogen-free) medium with or without 0.5 M NaCl. ∆AGP was included as a control of excretion of organic acids. ∆AGP cells were grown in BG11C without NaCl and transferred to BG11_0_C without NaCl. (A) Extracellular pyruvate and (B) 2-oxoglutarate levels normalized to culture cell density. Data are means ±SEM from three biological replicates. (This figure is available in colour at *JXB* online.)

Finally, glycogen-deficient mutants have been described to excrete organic acids to the growth medium under nitrogen deficiency, mainly pyruvate and 2-oxoglutarate (Carrieri *et al.*, 2012; Gründel *et al.*, 2012) (∆AGP in Fig. 9). In the culture medium of the SGLG36 strain, excreted pyruvate reached concentrations (normalized to culture cell density) of up to 0.14±0.04 mM 48 h after the removal of NaCl and nitrogen. However, when NaCl was not removed, pyruvate was not excreted to the culture medium (Fig. 9A). Similarly, 2-oxoglutarate accumulated in the medium of SGLG36 cultures when they were deprived of nitrogen, but only in the absence of NaCl, to levels similar to those found in ∆AGP cultures (0.19±0.06 and 0.18±0.03 mM, respectively; Fig. 9B). Thus, considerable amounts of organic acids are found in the culture medium only when carbon flux towards glycogen or GG synthesis is blocked.

All these results demonstrate that the distinctive phenotypes related to nitrogen deprivation of the glycogen-deficient mutants are not a consequence of the absence of glycogen synthesis by glycogen synthases, but are due to a blockage of the carbon flux. Thus, the existence of an alternative metabolic sink, such as GG synthesis, is sufficient to suppress the phenotypes described for glycogen-deficient mutants.

## Discussion

The use of cyanobacteria to produce biofuels or high-value chemicals is currently an area of great interest. This has led to the need for a better understanding of central metabolic processes and the development of metabolic engineering platforms with enhanced capability to improve the production of valuable compounds. One of the main strategies is to increase the availability of intermediary metabolites by eliminating competing routes. In this scenario, strains lacking the glycogen synthesis pathway have been used as a genetic background for the synthesis of several chemical commodities (Qi *et al.*, 2013; Jacobsen and Frigaard, 2014; Li *et al.*, 2014; van der Woude *et al.*, 2014; Namakoshi *et al.*, 2016), with variable results (Davies *et al.*, 2014; Veetil *et al.*, 2017; David *et al.*, 2018). In addition, the lack of this metabolic sink produces undesirable phenotypes in the mutants, which are apparent mainly under high light, light/dark cycling, or nutrient-deprivation conditions (Gründel *et al.*, 2012; Holland *et al.*, 2016). Understanding the causes behind these phenotypes will provide tools for the design of new metabolic strategies to enhance the use of these strains.

**Fig. 10.**
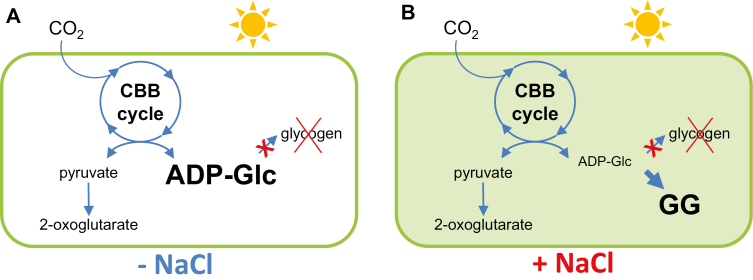
NaCl acts to relieve the blockage in carbon flux. (A) Blockage of the flux of fixed carbon towards glycogen by deleting glycogen synthases leads to a toxic accumulation of ADP-Glc. (B) Culture in NaCl-containing medium induces the synthesis of the osmolite glucosylglycerol (GG) from ADP-Glc, providing an alternative carbon sink and restoring metabolic carbon flux and cell viability. (This figure is available in colour at *JXB* online.)

In this work, we generated a *Synechocystis* mutant that lacks both of the glycogen synthases and accumulates large amounts of ADP-Glc. This accumulation is due to an imbalance in the synthesis and consumption of this metabolite. *In vitro*, the synthesis of ADP-Glc by AGP is reversible; however, hydrolysis of the released inorganic pyrophosphate by inorganic pyrophosphatase makes this reaction irreversible in the cell (Ballicora *et al.*, 2003). When mutant cells were grown in standard BG11C medium, there was no additional metabolic fate for the ADP-Glc and it accumulated to levels that were fatal to the cell. The presence of NaCl in the culture medium provided an alternative destination for ADP-Glc in the synthesis of GG, which restored cell viability (Fig. 10).

Accumulation of toxic amounts of ADP-Glc has been described in the plant *Arabidopsis thaliana* (Ragel *et al.*, 2013). In this case, lack of starch synthases (SS) 3 and 4, required for proper initiation of the starch granule, hampers the effective consumption of ADP-Glc, leading to the accumulation of this metabolite and pleiotropic phenotypes including growth retardation and impaired photosynthesis.

In *Escherichia coli*, an additional route for ADP-Glc consumption is its hydrolysis by an adenosine diphosphate sugar pyrophosphatase (AspP) (Moreno-Bruna *et al.*, 2001). This could explain the existence of viable *E. coli* glycogen synthase mutants in the presence of *glgC*. A similar reaction has been proposed to occur in *A. thaliana* (Rodriguez-Lopez *et al*., 2000). However, in *A. thaliana*, this activity may be not enough to decrease ADP-Glc to non-toxic levels in *ss3/ss4* mutants. To date, no enzyme with ADP-Glc hydrolase activity has been described in cyanobacteria.

The accumulation of moderate levels of ADP-Glc does not necessarily produce an apparent phenotype. This is the case in Arabidopsis mutants lacking SS4 (Crumpton-Taylor *et al.*, 2013) or the proteins PROTEIN TARGETING TO STARCH 2 and 3 (Seung *et al.*, 2017). This suggests that cells might be able to tolerate relatively high levels of ADP-Glc before it becomes toxic, and it could also explain why viable *glgA* mutants have been described in several cyanobacteria. In marine cyanobacteria, such as *Synechococcus* sp. PCC 7002, GG synthesis competes with glycogen synthesis for the ADP-Glc pool in standard growth conditions (Guerra *et al.*, 2013; Xu *et al.*, 2013), thus avoiding ADP-Glc accumulation. The situation is different in the freshwater cyanobacterium *Synechococcus elongatus* PCC 7942 grown in standard BG11. In this case, a viable mutant lacking glycogen synthase activity has been reported, although ADP-Glc is not redirected to GG synthesis (Suzuki *et al.*, 2010). In addition, a viable *glgA1/glgA2* double mutant has been described in *Synechocystis* (Gründel *et al.*, 2012). These observed differences may be due to differences in growth conditions or genetic backgrounds, or even to the fortuitous selection of a clone with a reduced rate of ADP-Glc synthesis, as no data on ADP-Glc content were reported.

When the SGLG36 strain was grown in NaCl-free medium, ADP-Glc synthesized by AGP could not be used for glycogen or GG synthesis (Fig. 4C, D). This increasing pool of unmetabolized ADP-Glc (up to five times the amount of total free adenine nucleotides) represents a pool of sequestered ADP that is not available for cellular metabolism. However, absolute levels of free ADP did not decrease (Fig. 7B), probably due to the generation of ADP at the expense of ATP and AMP by adenylate kinase, and by *de novo* AMP synthesis (Fig. 7C). Thus, the pool of total adenine nucleotides increased due to the AMP pool (Fig. 7B), which resulted in a relative decrease of the ATP and ADP pools and hence a decrease of the AEC (Fig. 7A). Furthermore, the elevated requirement for ATP for the *de novo* synthesis of AMP (eight ATP equivalents for AMP synthesis from ribose-5-phosphate) probably contributed to intensifying the decrease in AEC to values incompatible with cell viability (Chapman *et al.*, 1971).

Most of the metabolic processes in the cell may be affected directly or indirectly by the imbalance in the adenine nucleotide pools, either by an energy shortage for anabolic processes or by interference in the allosteric regulation of enzymes. Alteration in the photosynthetic capacity of SGLG36 (Fig. 6) may be both a consequence and a cause of the imbalance in the adenine nucleotide pools after NaCl removal. The decrease in PSII operating efficiency, measured as Y(II), can be attributed to a more reduced state of the plastoquinone pool (Stirbet *et al.*, 2019). However, the negative effect of ADP-Glc accumulation not only affected the effective photosynthetic capacity of SGLG36, but subsequently affected its maximum capacity, which suggests specific damage to the photosynthetic machinery itself.

It is notable that SGLG36 was strictly salt dependent (Supplementary Fig. S2D, Fig. 4A, B); thus, loss of both glycogen synthases in *Synechocystis* transforms this freshwater cyanobacterium into a halodependent organism. This feature could have potential biotechnological value in the selection of modified strains in which efficient redirection of the carbon flux towards metabolites of interest prevents the accumulation of ADP-Glc and allows this strain to grow in conditions with lower salt content.

The SGLG36 mutant may also be a useful tool to address questions related to the basic physiology of *Synechocystis*, such as the role of glycogen in the response to nitrogen deficiency. As previously indicated, several studies have described alterations in the response to nitrogen deprivation in mutants incapable of glycogen synthesis. In this work, we demonstrate that the main phenotypes ascribed to these mutants (immediate cessation of growth, non-bleaching, and excretion of organic acids) are not ultimately a consequence of glycogen synthesis deficiency but a result of the metabolic carbon flux blockage: when the carbon flux is redirected to the synthesis of GG in the presence of NaCl, the response to nitrogen deprivation is restored (Figs 8 and 9, Supplementary Fig. S5). We propose that the reason for the blockage of the nitrogen-deficiency response in these mutants is alterations at the metabolite level, either the exhaustion or accumulation of one or more metabolites, as has previously been suggested (Gründel *et al.*, 2012; Hickman *et al.*, 2013).

In conclusion, our work demonstrates that manipulation of the metabolic carbon flux transforms a freshwater cyanobacterium into an organism that strictly requires NaCl for its survival—a feature that has potential biotechnological interest. This phenotype is due to a toxic accumulation of ADP-Glc, which severely affects the energy status of the cell. Finally, we have demonstrated that metabolic carbon flux, and not glycogen synthesis itself, is required for a correct metabolic response to nitrogen deprivation in *Synechocystis*.

## Supplementary data

Supplementary data are available at *JXB* online.

**Table S1.** Sequences of oligonucleotides used in this work.

**Fig. S1.** Schematic representation of the mutant strains employed in this work.

**Fig. S2.** NaCl is required to obtain a fully segregated mutant lacking both glycogen synthases while keeping *glgC*.

**Fig. S3.** NaCl exerts a dose-dependent effect on the growth rescue of the SGLG36 strain.

**Fig. S4.** Levels of adenine nucleotides are altered in the SGLG36 mutant after salt and nitrogen removal.

**Fig. S5.** Pigment degradation in response to nitrogen deprivation.

erz559_suppl_Supplementary_Table_S1_Figures_S1_S5
