# Supplementary material for: Lethality caused by ADP-glucose accumulation is suppressed by salt-induced carbon flux redirection in cyanobacteria
Source: J Exp Bot. 2019 Dec 20;71(6):2005–17. doi: 10.1093/jxb/erz559 (PMC7242066; doi:10.1093/jxb/erz559)
Supplement: erz559_suppl_Supplementary_Table_S1_Figures_S1_S5 [file erz559_suppl_supplementary_table_s1_figures_s1_s5.pdf]

SUPPLEMENTARY DATA

Article title:

Lethality caused by ADP-Glucose accumulation is suppressed by salt-induced carbon flux redirection in cyanobacteria

Authors:

Sandra Díaz-Troya, Miguel Roldán, Manuel J. Mallén-Ponce, Pablo Ortega-Martínez and Francisco J. Florencio.

Institution and e-mail address:

Sandra Díaz-Troya: Instituto de Bioquímica Vegetal y Fotosíntesis, Universidad de Sevilla-CSIC, 41092 Sevilla, Spain. sdtroya@us.es. Tel. +34 954489501 Ext. 909668

Miguel Roldán: Instituto de Bioquímica Vegetal y Fotosíntesis, Universidad de Sevilla-CSIC, 41092 Sevilla, Spain. mrgalvez@ibvf.csic.es

Manuel J. Mallén-Ponce: Instituto de Bioquímica Vegetal y Fotosíntesis, Universidad de Sevilla-CSIC, 41092 Sevilla, Spain. mmallen@us.es

Pablo Ortega-Martínez: Instituto de Bioquímica Vegetal y Fotosíntesis, Universidad de Sevilla-CSIC, 41092 Sevilla, Spain. pabortmar1@alum.us.es

Francisco J. Florencio: Instituto de Bioquímica Vegetal y Fotosíntesis, Universidad de Sevilla-CSIC, 41092 Sevilla, Spain. floren@us.es. Tel. +34 954489509.

Corresponding authors: Francisco J. Florencio and Sandra Díaz-Troya

This document contains:

Supplementary Table S1

Supplementary Fig. S1-5

Supplementary Table S1. Sequences of oligonucleotides used in this work.

Primer name	Sequence
**glgC_NdeI_5'**	CCGGCATATGGTGAAACGTGTCTTAGC
**glgC_NotI_3'**	GCGGCCGCTAGATTACCGTGCCG
**glgC_UP_5'**	CCGTTTACTAATGGCATCAACGGCG
**glgC_DO_3'**	GCGATCCTCCGGCTTAATCTGGGAG
**glgA1_UP_5'**	GCCTTATTCTGTTGCTACGTCAATG
**glgA1_BamHI_UP2_3'**	CAGTGGAGCGGGATCCGGGTTCA
**glgA1_BamHI_DO2_5'**	AAATCCTGAACCCGGATCCCG
**glgA1_DO_3'**	TAGAATGAAGCTGGAAATCGGCTC
**glgA1_1F**	CTCAATTAGTGGAATAACGACGGT
**glgA1_1R**	AGAATTGGGGTCGGCAGTGG
**glgA2_UP_5'**	GCGGCGCTTGGTATTTGTGGAAG
**glgA2_BamHI_UP2_3'**	CCAACTGCTTTGACGGATCCTCTG
**glgA2_BamHI_DO2_5'**	ACTAGTTCAGAGGATCCGTCAAAG
**glgA2_DO_3'**	CCTGGGTAGGGTTATGGCTTTC
**sll1815_1F**	GGTTACATGGACAAGGGAGAAT
**sll1815_1R**	GGTTACCGTCAAGGGAGTAAAG
**sll1823_1F**	GAGGCATTGGTCCCACCTAC
**sll1823_1R**	CCGCAATAGGGTTGGAGGAG
**sll1566_1F**	GAGTGATCCAGGCAATGTCG
**sll1566_1R**	CGTAGTCTCAAGCCGGTGG

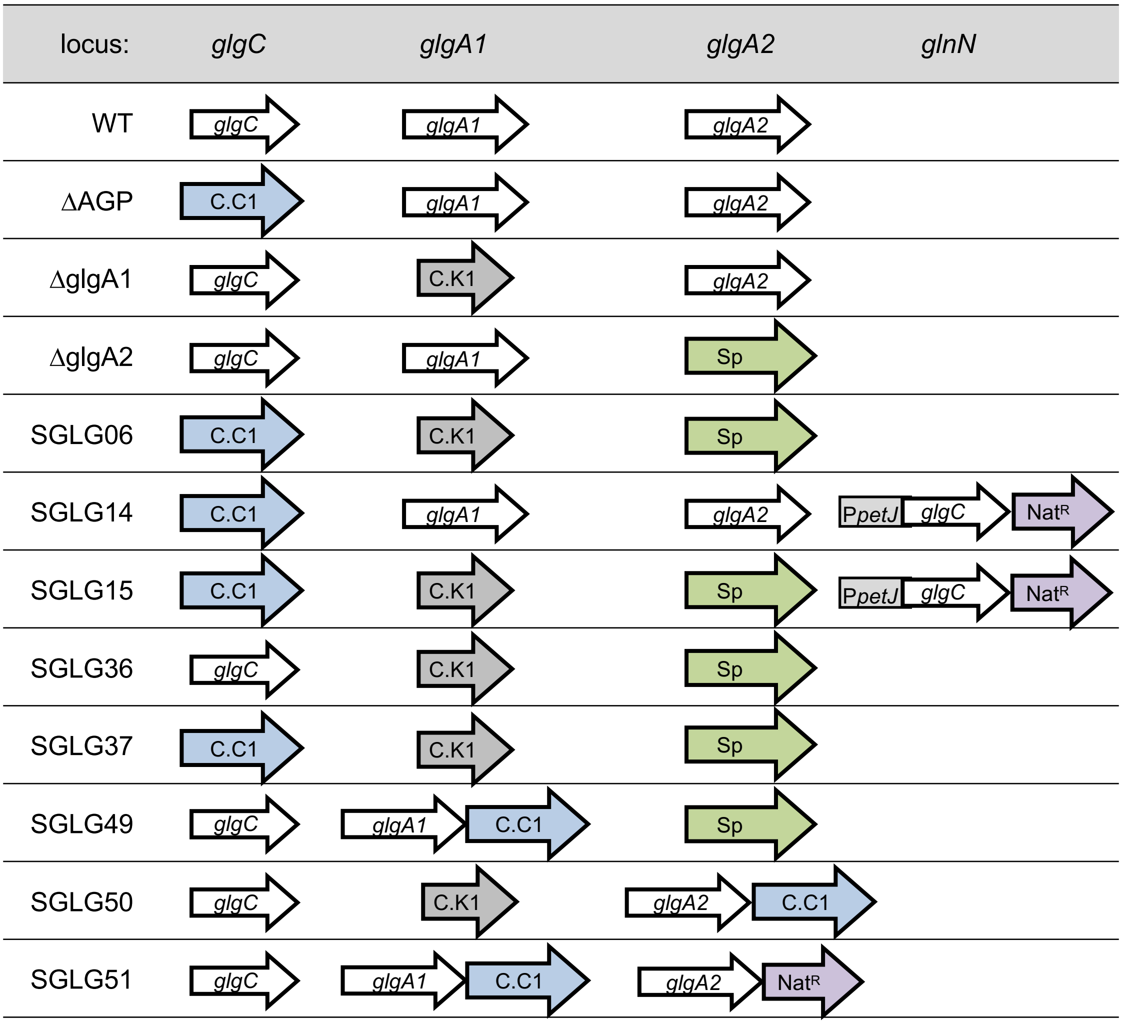

Supplemental Fig. S1. Schematic representation of the mutant strains employed in this work.

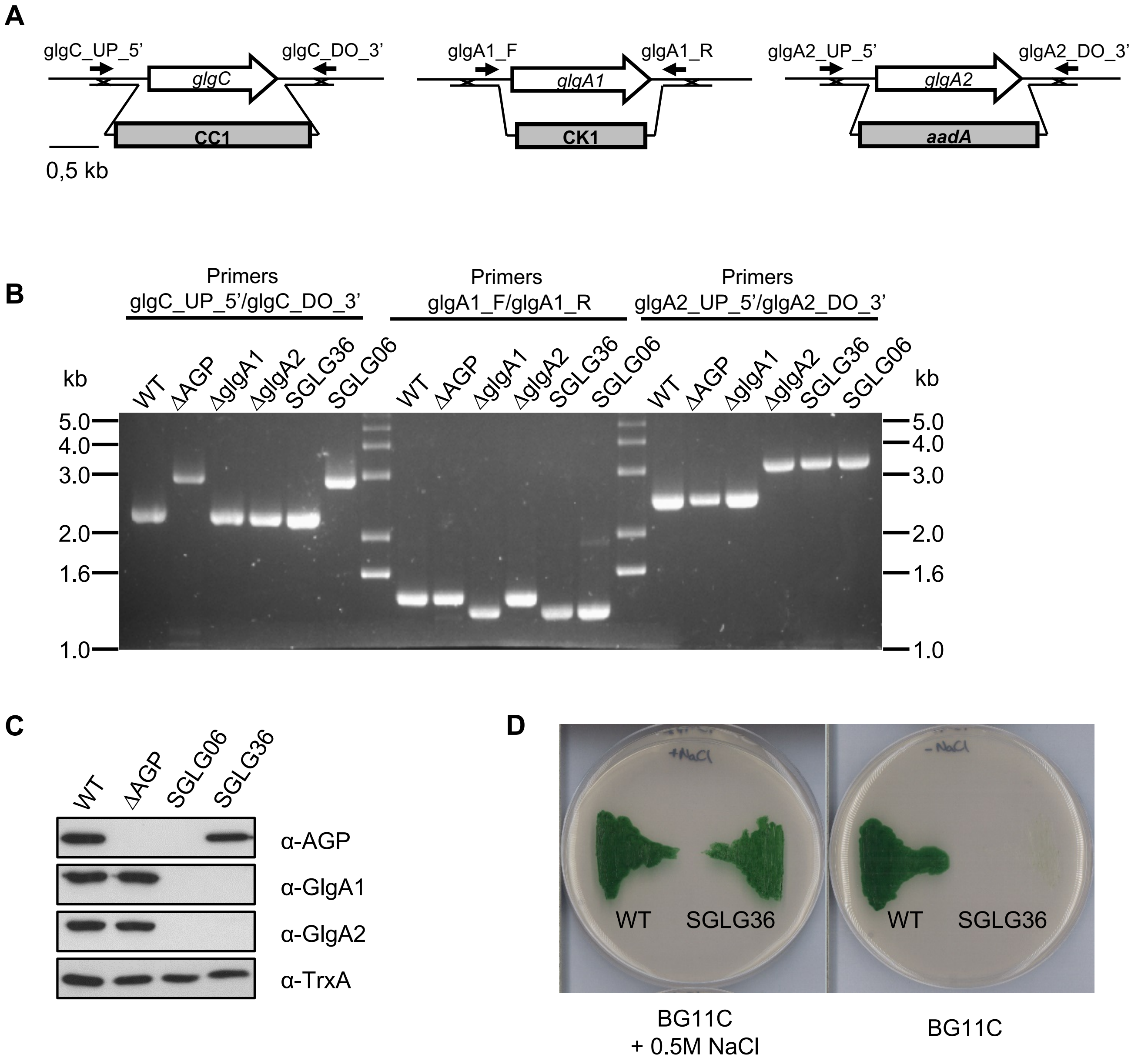

Supplemental Fig. S2. NaCl is required to obtain a fully segregated mutant lacking both glycogen synthases while keeping *glgC*. (A) Schematic representation of the strategy for the deletion of *glgC*, *glgA1* and *glgA2*, including primers used in *B*. (B) PCR to validate the complete segregation of the different mutant strains. (C) Western blot analysis of total soluble extracts from WT, ΔAGP, SGLG06 and SGLG36 cells with the indicated antibodies. (D) Growth of WT and SGLG36 cells in BG11C plates with and without 0.5 M NaCl.

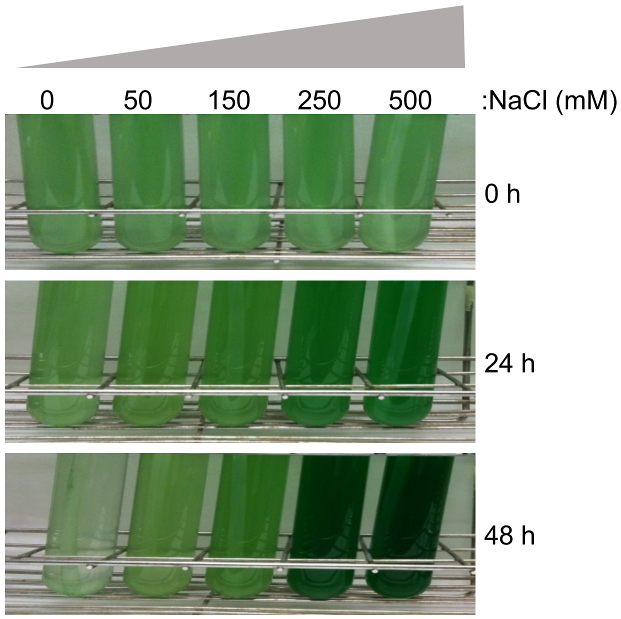

Supplemental Fig. S3. NaCl exerts a dose-dependent effect on the growth rescue of SGLG36 strain. SGLG36 cells were grown in NaCl-supplemented media and transferred to media supplemented with increasing amounts of NaCl. Photographs of the cultures were taken at different times of cultivation.

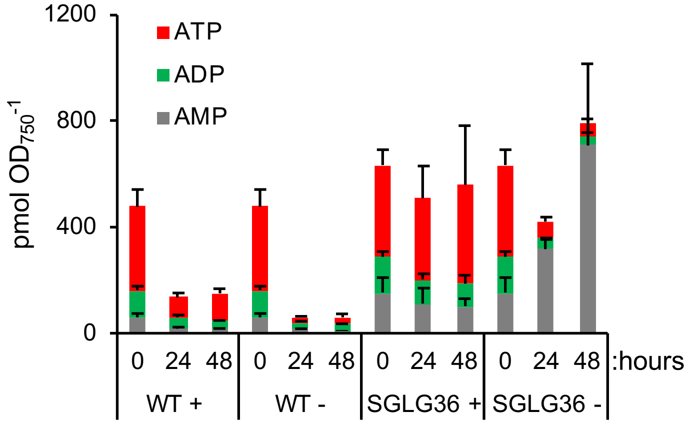

Supplemental Fig. S4. Levels of adenine nucleotides are altered in SGLG36 mutant after salt and nitrogen removal. WT and SGLG36 cells grown in BG11C with NaCl were transferred to $BG11_0C$ (nitrogen free) media with (+) or without (-) 0.5 M NaCl. Quantification of ATP, ADP and AMP at the indicated times. Data are means ± SEM from 2 biological replicates for WT and 3 for SGLG36.

.

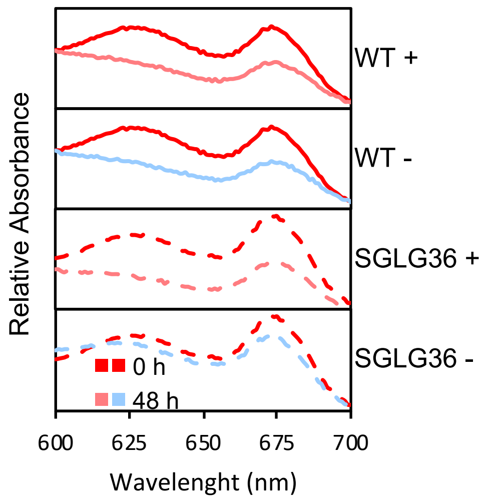

Supplemental Fig. S5. Pigment degradation in response to nitrogen deprivation. WT and SGLG36 cells were grown in BG11C (17.6 mM $NaNO_3$) containing 0.5 M NaCl and transferred to $BG11_0C$ (nitrogen free) media with (+) or without (-) 0.5 M NaCl. Whole cell absorbance spectra were measured before and 48h after nitrogen deprivation.
